# Supplementary material for: Urban air pollution and emergency department visits related to central nervous system diseases
Source: PLoS One. 2022 Jun 27;17(6):e0270459. doi: 10.1371/journal.pone.0270459 (PMC9236246; doi:10.1371/journal.pone.0270459)

**S1 File**

**Urban air pollution and emergency department visits related to central nervous system diseases.**

Anna O. Lukina^1^, Brett Burstein^2,3^, Mieczysław Szyszkowicz^1^*

^1^-Environmental Health Science and Research Bureau, Health Canada, Ottawa, Canada.

^2^- Division of Pediatric Emergency Medicine, Department of Pediatrics, Montreal Children's Hospital, McGill University Health Centre, Montreal, Quebec, Canada.

^3^- Department of Epidemiology, Biostatistics and Occupational Health, McGill University, Montreal, Quebec, Canada.

This section contains two tables (S1 and S2) and five figures (S1-S5). The figure is a map to show the associations determined for all constructed models. The estimated values (*Beta*, SE *Beta* {standard error}) are located at [https://github.com/szyszkowiczm/NERVEToronto](https://github.com/szyszkowiczm/ResultsToronto).

S1 Table: Descriptive statistics on the number of ED visits related to nervous system diseases in total and based on daily counts during the entire study.

| **Variables** | **ED visits** | **%** | **Min** | **Q1** | **Median** | **Mean** | **Q3** | **Max** |
| --- | --- | --- | --- | --- | --- | --- | --- | --- |
| All | 140,511 | - | 8 | 26 | 32 | 32.7 | 39 | 66 |
| Female | 83,602 | 59.5 | 3 | 15 | 19 | 19.5 | 24 | 46 |
| Male | 56,909 | 40.5 | 1 | 10 | 13 | 13.3 | 16 | 33 |
| Warm All | 72,880 | - | 10 | 27 | 33 | 33.2 | 39 | 66 |
| Warm Female | 43,432 | 59.6 | 4 | 15 | 20 | 19.8 | 24 | 46 |
| Warm Male | 29,448 | 40.4 | 1 | 10 | 13 | 13.4 | 16 | 33 |
| Cold All | 67,631 | - | 8 | 26 | 32 | 32.3 | 38 | 61 |
| Cold Female | 40,170 | 59.4 | 3 | 15 | 19 | 19.2 | 23 | 41 |
| Cold Male | 27,461 | 40.6 | 1 | 10 | 13 | 13.1 | 16 | 29 |
| Age 0-10 All | 4,292 | - | 0 | 0 | 1 | 1.0 | 2 | 7 |
| Age 0-10 Female | 1,978 | 46.1 | 0 | 0 | 0 | 0.5 | 1 | 5 |
| Age 0-10 Male | 2,314 | 53.9 | 0 | 0 | 0 | 0.5 | 1 | 7 |
| Age 11-60 All | 93,633 | - | 4 | 17 | 21 | 21.8 | 26 | 45 |
| Age 11-60 Female | 57,456 | 61.4 | 2 | 10 | 13 | 13.4 | 16 | 31 |
| Age 11-60 Male | 36,177 | 38.6 | 0 | 6 | 8 | 8.4 | 11 | 25 |
| Age 60+All | 42,586 | - | 0 | 7 | 10 | 9.9 | 12 | 24 |
| Age 60+ Female | 24,168 | 56.8 | 0 | 4 | 5 | 5.6 | 7 | 17 |
| Age 60+ Male | 18,418 | 43.2 | 0 | 3 | 4 | 4.3 | 6 | 14 |

Notes: Column labelled as “ED visits” shows the number of ED visits related to nervous system diseases, Warm season: April – September, Cold season: October – March, Min – minimum, Max –maximum, Q1-25^th^ percentile, Q3-75^th^ percentile-all of those values are based on daily counts.

S2 Table: Descriptive statistics on all five air pollutants studied, calculated air quality indices (based on ground level O_3_ collected at a 24-hr average and 8-hr average), and meteorological conditions (ambient temperature and relative humidity) collected from seven monitoring stations in Toronto, Canada between April 1, 2004 and December 31, 2015.

| **Factors** | **Min** |  | **Q1** | **Median** | **Mean** | **Q3** | **Max** | **IQR** |
| --- | --- | --- | --- | --- | --- | --- | --- | --- |
| PM_2.5_ (μg/m^3^) | 0.1 |  | 4.7 | 7.1 | 8.9 | 11.2 | 65.5 | 6.5 |
| NO_2_ (ppb) | 3.2 |  | 11.1 | 15.0 | 16.1 | 19.9 | 59.8 | 8.8 |
| O_3_ (ppb) | 1.7 |  | 16.8 | 23.0 | 23.5 | 29.6 | 62.1 | 12.8 |
| O_3_H8 (ppb) | 9.0 |  | 33.0 | 41.0 | 43.7 | 52.0 | 107.0 | 19.0 |
| SO_2_ (ppb) | -0.5 |  | 0.5 | 1.0 | 1.4 | 1.7 | 12.0 | 1.2 |
| CO (ppm) | 0.0 |  | 0.2 | 0.2 | 0.3 | 0.3 | 1.1 | 0.1 |
| AQHI (number) | 1.0 |  | 2.4 | 2.9 | 3.0 | 3.4 | 7.6 | 1.0 |
| AQHI-x (number) | 1.6 |  | 3.6 | 4.2 | 4.4 | 5.1 | 10.3 | 1.5 |
| Temperature (^o^C) | -22.2 |  | 1.7 | 10.0 | 9.5 | 18.4 | 31.2 | 16.7 |
| Relative Humidity (%) | 31.7 |  | 63.9 | 70.9 | 70.7 | 78.2 | 98.8 | 14.3 |

Notes: Min-minimum, Max –maximum, Q1-25^th^ percentile, Q3-75^th^ percentile, IQR=Q3-Q1.

S1 Fig: Number of associations between 18 strata (classified by patients’ sex and age, and seasonality) (rows) and five studied air pollutants and air quality health indices (one with a 24-hour average of ground-level O_3_ expressed as AQHI and another one with an 8-hour maximum ground-level O_3_ expressed as AQHIX) (columns). The ICD-10 codes are G00-99 for all nervous system disorders. All 15 time lags (0-14 days) following exposure are combined. For visual representation: 0 (green) colour represents other than positive statistically significant associations.


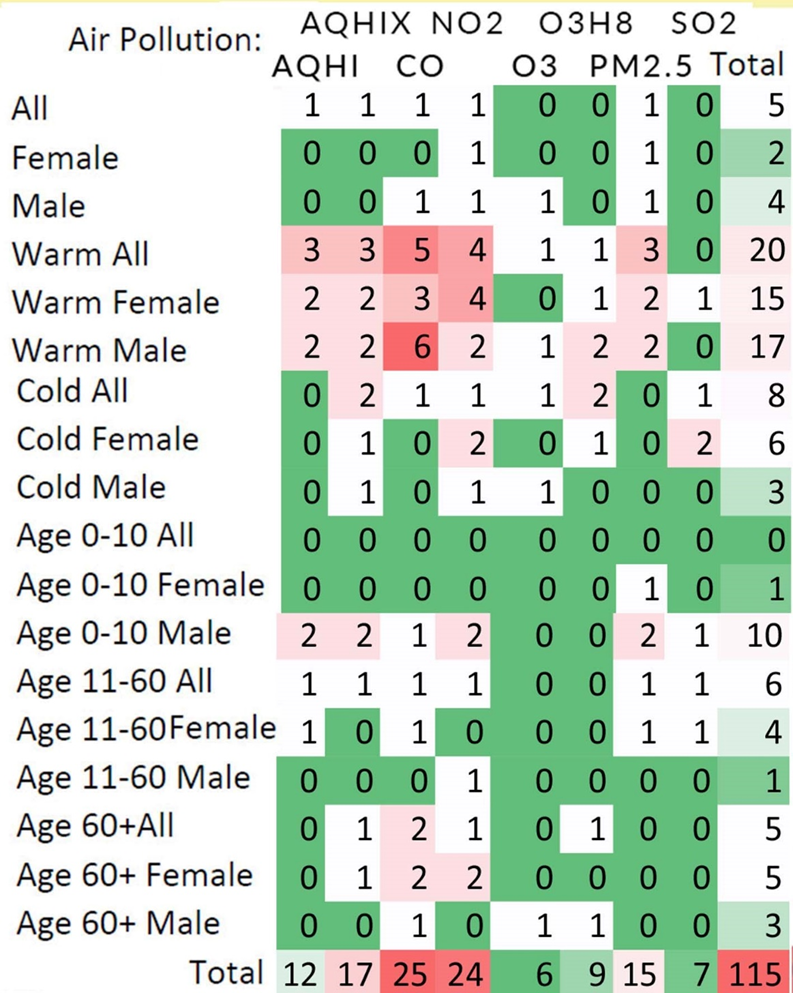


S2 Fig: Total frequencies of all associations for ambient air pollutants and the number of ED visits related to nervous system diseases (G00-G99). Eighteen strata (classified by patients’ sex and age, as well as cold vs. warm seasons) examined and arranged in rows and 15 lags (expressed as days) are arranged in columns. For visual representation: 0 (green) colour represents other than positive statistically significant associations.


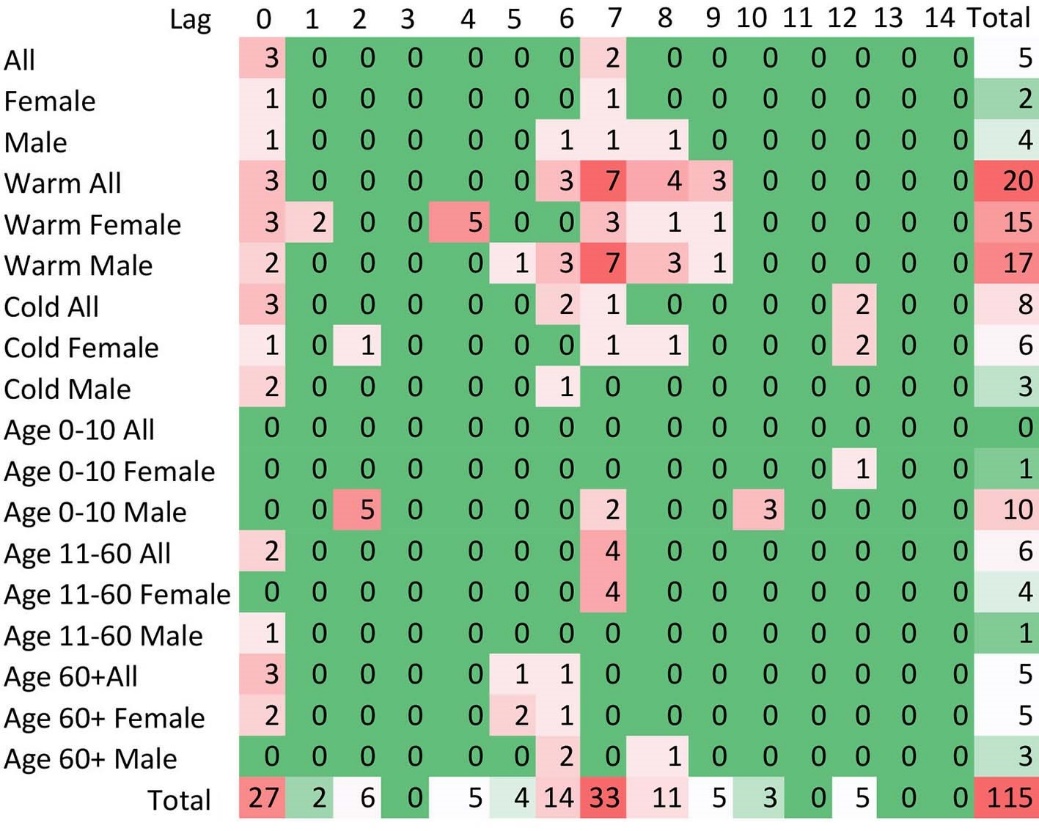


S3 Fig: Total frequencies for all associations: five air pollutants + two air quality health indices (rows) and 15 time lags of 0-14 days (columns), between exposure to urban air pollutants levels and the number of ED visits for nervous system diseases (G00-G99) in Toronto, Canada, between April 1, 2004 and December 31, 2015. For visual representation: 0 (green) colour represents other than positive statistically significant associations.


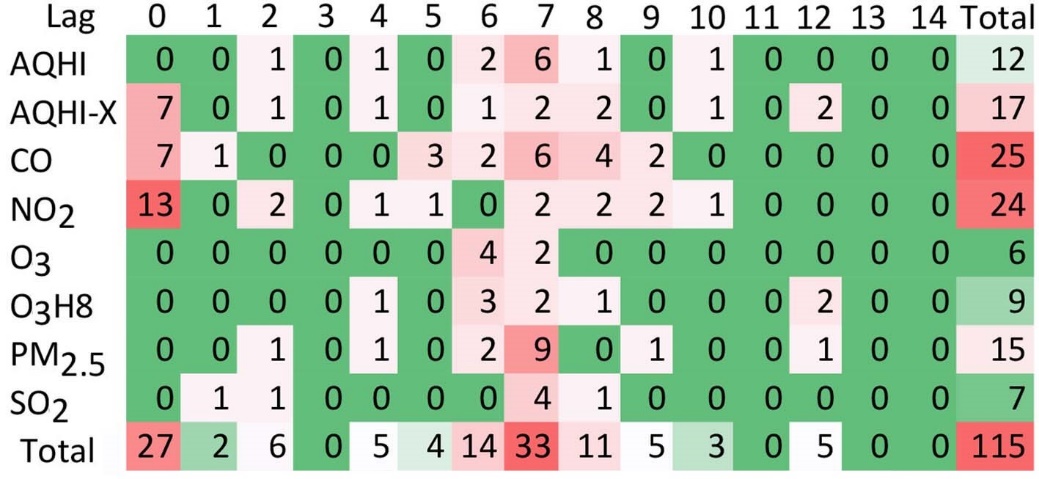


S4 Fig: A map of numerical results for 18 strata (rows) and 15 time lags (columns). 0/white – no associations, and statistically significant: -1/green – negative, 1/red – positive. The results are grouped by all five ambient air pollutants and AQHI and AQHIX indices. The ICD-10 codes are G00-G99 (all nervous system diseases) in relation to each air pollutant. The corresponding numerical values are also available at [https://github.com/szyszkowiczm/NERVEToronto](https://github.com/szyszkowiczm/ResultsToronto) in the file ”[NERVEICDG0099Toronto.csv](https://github.com/szyszkowiczm/ResultsToronto/blob/master/NERVEICDG4047Toronto.csv)”


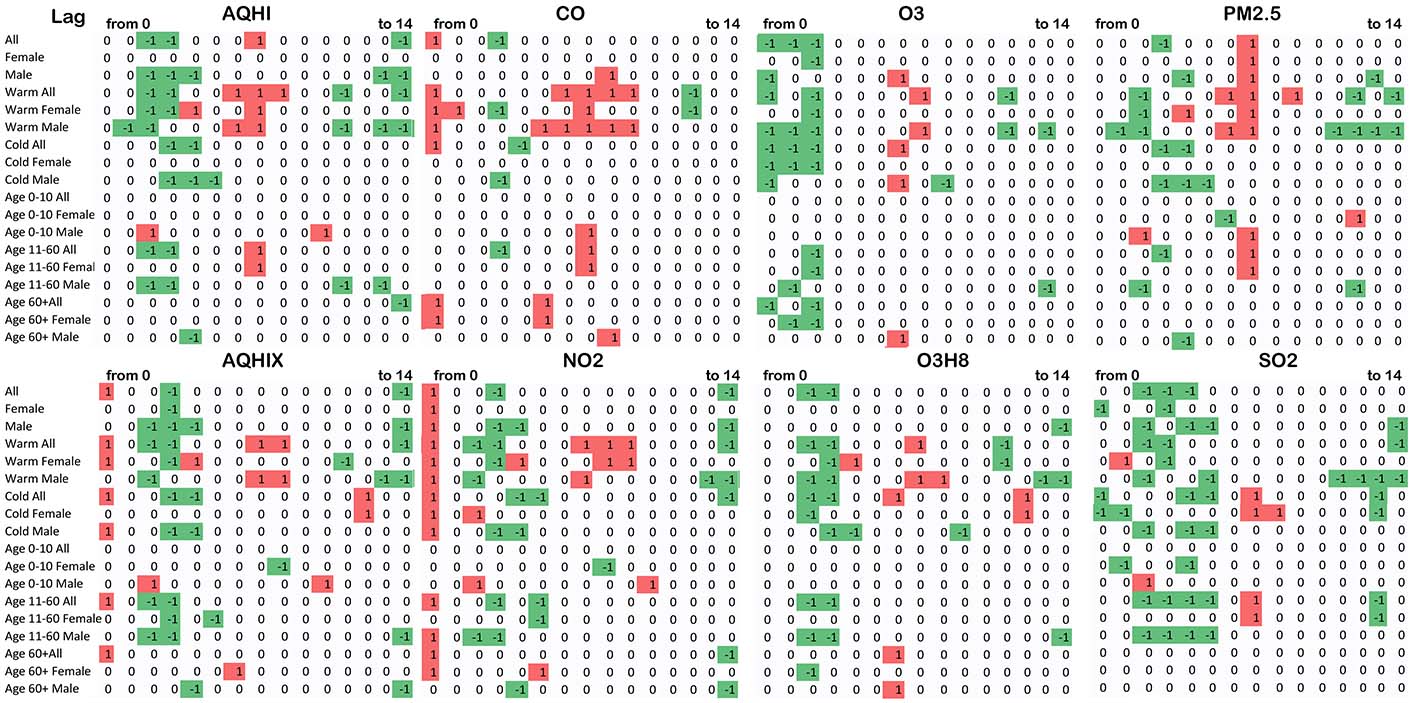


S5 Fig: A map of numerical results for 18 strata (rows) and 15 time lags (columns). 0/white – no associations, and statistically significant: -1/green – negative, 1/red – positive. The results are grouped by all five ambient air pollutants and AQHI and AQHIX indices. The ICD-10 codes are G40-47 (episodic and paroxysmal disorders) in relation to each air pollutant. The corresponding numerical values are also available at [https://github.com/szyszkowiczm/NERVEToronto](https://github.com/szyszkowiczm/ResultsToronto) in the file ” “[NERVEICDG4047Toronto.csv](https://github.com/szyszkowiczm/ResultsToronto/blob/master/NERVEICDG4047Toronto.csv)”


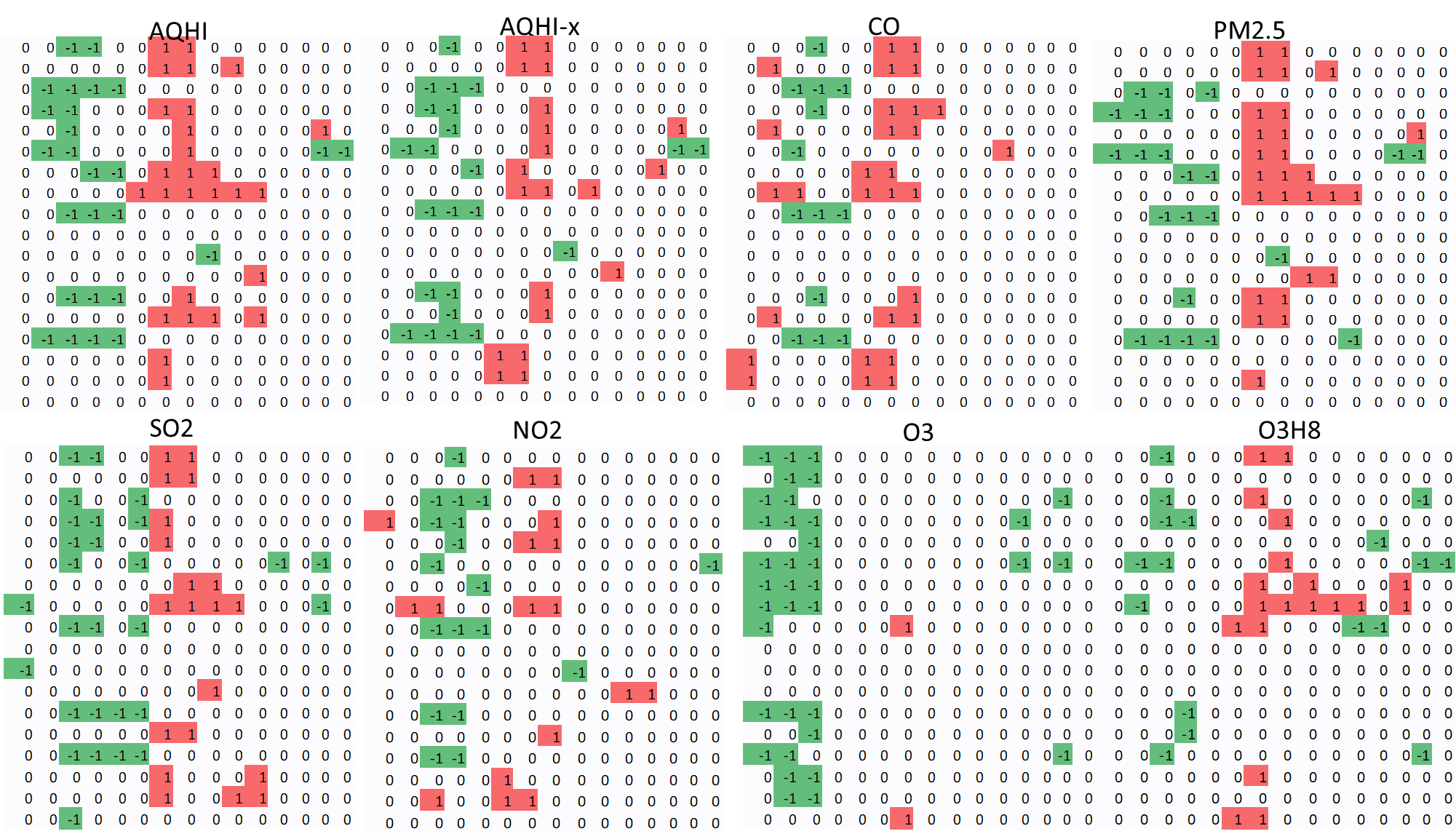

Supplement: S1 File — (DOCX) [file pone.0270459.s001.docx]
