# Supplementary material for: Urban air pollution and emergency department visits related to central nervous system diseases
Source: PLoS One. 2022 Jun 27;17(6):e0270459. doi: 10.1371/journal.pone.0270459 (PMC9236246; doi:10.1371/journal.pone.0270459)
Supplement: Attachment — Submitted filename: PONE-D-21-18477-Rebuttal Letter-Response to Reviews.docx [file pone.0270459.s002.docx]

Dear Dr. Xu,

Thank you again for your time and effort in handling our manuscript. We are grateful to the reviewers for their comments, which have enabled us to improve our manuscript. Below, we address the points raised by each reviewer (in black text) and provide details of how we have responded to each point (in blue text). We have submitted a tracked version of our revised manuscript as well as an untracked, clean version.

Sincerely,

Dr. Mieczysław Szyszkowicz

(new email: mietek.szyszkowicz@hc-sc.gc.ca)

Reviewer's Responses to Questions (reviewer comments (in black text) and our responses (in blue text):

**Comments to the Author**

1. Is the manuscript technically sound, and do the data support the conclusions?

The manuscript must describe a technically sound piece of scientific research with data that supports the conclusions. Experiments must have been conducted rigorously, with appropriate controls, replication, and sample sizes. The conclusions must be drawn appropriately based on the data presented.

Reviewer #1: No

Reviewer #2: Partly

2. Has the statistical analysis been performed appropriately and rigorously?

Reviewer #1: Yes

Reviewer #2: No

3. Have the authors made all data underlying the findings in their manuscript fully available?

The [PLOS Data policy](http://www.plosone.org/static/policies.action#sharing) requires authors to make all data underlying the findings described in their manuscript fully available without restriction, with rare exception (please refer to the Data Availability Statement in the manuscript PDF file). The data should be provided as part of the manuscript or its supporting information, or deposited to a public repository. For example, in addition to summary statistics, the data points behind means, medians and variance measures should be available. If there are restrictions on publicly sharing data—e.g. participant privacy or use of data from a third party—those must be specified.

Reviewer #1: No

Reviewer #2: No

4. Is the manuscript presented in an intelligible fashion and written in standard English?

PLOS ONE does not copyedit accepted manuscripts, so the language in submitted articles must be clear, correct, and unambiguous. Any typographical or grammatical errors should be corrected at revision, so please note any specific errors here.

Reviewer #1: Yes

Reviewer #2: No

5. Review Comments to the Author

Please use the space provided to explain your answers to the questions above. You may also include additional comments for the author, including concerns about dual publication, research ethics, or publication ethics. (Please upload your review as an attachment if it exceeds 20,000 characters)

Reviewer #1: Lukina et al. examined the associations between short-term air pollution and emergency department visits related to neurological disorders. The manuscript needs more work in the Introduction, Methods, and Results.

Thank you very much for your positive comments and constructive feedback.

1. Introduction. As mentioned in the Introduction, several studies have examined the associations between air pollution and neurological outcomes. The Introduction would benefit from discussing the maturation of the current literature, the research gaps, and the need to conduct the present study.

Response: As suggested, we have expanded our introduction section by discussing the current literature, the research gaps and adjusting our study objectives for better read (lines: 109-122-marked version).

2. Methods. Please list out all the CNS disorders included in this study and their corresponding ICD codes.

Response: We have included all 11 diagnostic sub-codes in the text (lines: 154-162-marked version).

3. Statistical models. Instead of describing the slope and standard errors, it would be clear to write out the formula of the conditional quasi-Poisson regression. It would also be helpful to include the statistical testing and measures of effects.

Response: As requested, we provided the model presentation (using R statistical software). It was shown (Ref: Armstrong et al. 2014) that such realization is equivalent to conditional logistic regression realized in the standard case-crossover method. We calculated relative risks with associated 95% confidence intervals for all models and the numerical results are presented at the location: <https://github.com/szyszkowiczm/NERVEToronto>. We did two analyses; for G00-G99 (all nervous system diseases) and G40-G47 separately, where G40-G47 (episodic and paroxysmal disorders) is 64% of all ED visits, but this group is more related to air pollution (publications on migraine, headache previously done by Szyszkowicz et al 2008, 2009a,b,c and many others). For this subgroup we see more (from 115 to 161) positive associations, especially consistent effect of ambient CO exposure for both G00-G99 and G40-G47.

Reference: Armstrong BG, Gasparrini A, Tobias A. Conditional Poisson models: a flexible alternative to conditional logistic case cross-over analysis. BMC Med. Res. Methodol. 2014; 14:122. doi: 10.1186/1471-2288-14-122.


4. Figure 1. Please write out the effect estimates in the main results. The figure is innovative yet confusing.

Response: As requested, we included three figures instead outlining by time lags, air pollutants/air quality health indexes, and strata classified by patients’ age and sex, and seasons (warm versus cold).

Reviewer #2: The authors conducted a case-crossover study in Canada on the associations between ED visits for CNS diseases and air pollution. Overall, the quality of this work needs to be improved, in both methodological and structural aspects. There are also weird grammars and expressions in text that need to be fixed by experts in this field. I listed my detailed comments as follows.

Thank you very much for your positive comments and constructive feedback. We have carefully gone throughout the manuscript and corrected / improved sentences (all changes tracked). We note that Reviewer #1 indicated that the writing is generally good.

1. Line 44, the aim of the study should be presented in a complete sentence.

Response: We reworded the objective of the study: “The present study investigated all possible associations between concentrations of air pollutants and the number of emergency department (ED) visits for nervous system disorders among people residing in Toronto, Canada.” (lines: 44-47-marked version).

2. Line 56, result description should accompany values with their significance.

Response: We expanded the results portion in abstract section: “For all ED visits (G00-G99) and for G40-G47, positive statistically significant associations were 115 and 161, respectively. For G40-G47, among females an increase in one interquartile range (IQR=0.1ppm) of ambient CO gives the following relative risks and 95%CIs [lag1: 1.019(1.004-1.033), lag6 (1.024(1.010-1.039)) and lag7 (1.022(1.007-1.036)]. Women older than 60 years of age were also affected by CO on lag0 (1.036(1.007-1.065)). Other pollutants, PM_2.5_ and SO_2_, and air indices were also associated with ED visits for episodic and paroxysmal disorders in lag6 and lag7.” (lines: 64-73-marked version).

3. Line 79, I would rather say PM, NO2 and O3 acquired most study interest.

Response: We reworded the sentence. However, we respectfully disagree with this reviewer on this point. In the statement, we refer to three major air pollutants that indeed have been studied quite a lot, because many epidemiological studies have consistently shown associated mortality and morbidity from exposure to major air pollutants. According to the World Health Organization (WHO), air pollution is the fifth-leading mortality health risks in the world and responsible to nearly 4.9 million premature deaths in 2017. Similar estimates were done by the Global Burden of Diseases (GBD), where both PM2.5 and O3 was responsible for 4.5 million premature deaths. We cannot eliminate the statement that major pollutants like PM2.5, O3 and NO2 pose a significant threat to human health. The reference already provided presents comprehensive estimate of all health outcomes related to such three air pollutants.

4. Line135, how was AQHI and AQHI-x calculated? To be exact.

Response: We included the formula: The AQHI values are generated using the following formula:

$$\boldsymbol{AQHI=}\frac{\boldsymbol{1000}}{\boldsymbol{10.4}}\boldsymbol{\times}\left( \boldsymbol{e}^{\boldsymbol{0.000537*O}\boldsymbol{3}} \right.\boldsymbol{+}\boldsymbol{e}^{\boldsymbol{0.000871*NO}\boldsymbol{2}}\boldsymbol{+}\boldsymbol{e}^{\boldsymbol{0.000487*PM}\boldsymbol{2.5}}\boldsymbol{-}\left. \boldsymbol{3} \right),$$

where the coefficients for air pollutants were estimated using mortality risks [25]. Lines: 183-185-marked version.

AQHIx is using the same three pollutants but ozone is used at an 8-hr average because it requires sunlight, which already mentioned in lines186-191-marked version.

5. Line 148, why and how to match cases with controls should be stated clearly.

Response: As requested, we expanded on such matter: “The time-stratified approach was applied to cluster the data using a calendar hierarchical structure, where days were nested in day of weeks, which further nested in months and then in years [28]. Such clusters grouped four or five days and were separated from each other. Controlling of time variables (i.e., any trends or fluctuations, etc.) was done according to the applied methodology as described previously [30].” (Lines: 206-211-marked version).

6. The generalized non-linear models are rarely used for case crossover designs, suggest to try the common conditional logistic regression models.

Response: We added the model description. We are using conditional Poisson regression. As Armstrong et al. 2014 presented, this approach is equivalent to the conditional logistic regression analysis.

Reference: Armstrong BG, Gasparrini A, Tobias A. Conditional Poisson models: a flexible alternative to conditional logistic case cross-over analysis. BMC Med. Res. Methodol. 2014; 14:122. doi: 10.1186/1471-2288-14-122.

7. How were metrological factors controlled in the models? The non-linear associations should be considered by natural splines or DLNM cross-basis patterns.

Response: We added the presentation of the used model (in R statistical software). Meteorological factors (ambient temperature and relative humidity) are presented as natural spline with three degrees of freedom. This study belongs to more general approach under assumption “Ambient air pollution may be damaging every organ and virtually every cell in the human body”. We still fully don’t know which health problems are related to ambient air pollution concentration levels. As the applied methodology allows relatively fast “scan” of the associations we mainly did this. Our findings are supported that for the subgroup (G40-G47-episodic and paroxysmal disorders) we observe more positive statistically significant associations (161 in total) versus more broad G00-G99-nervous system diseases, with 115 positive statistically significant associations. What was proposed (say distributed lag non-linear model or DLNM) can be sued to the detailed study, say migraine attacks.

8. What are the missing rates for air pollutants on both case/control days?

Response: We used the case-crossover (CC) design but didn’t realize it as case/control, rather daily count of events on the clusters. Such approach is (purely) equivalent to the CC method (described in details in Armstrong et al. 2014).

9. Line 157-158, suggest to change this expression. Also, if hundreds of models are performed, multiple testing is needed for type-I error.

Response: The numerical calculations were done in R statistical software using the package *GNM* with the option “quasipoisson”. The quasi-Poisson regression is realized to model an over dispersed count variable.

As in raised point 7, we realize “to scan” of the potential associations. The number of the correlations increase (from 115 to 161 positive statistically significant associations) if we used ED visits (G40-G47-episodic and paroxysmal disorders) with more health conditions already linked with ambient air pollution (assuming migraine attacks-39% as majority of cases). Also we applied the “quasi-Poisson” option which gives wider 95% confidence intervals. We listed all results, the proposed coloured heat map, which allows to identify the patterns of the associations. These associations can be studied more in details, say ambient air pollution concentration levels and number of ED visits for migraine attacks (from common (without aura) to classical (with aura) to unspecified migraines to complicated migraines/status migrainosus) among females.

In our approach, even the used data from the NACRS are daily counts and time-series type, we don’t have issues of the used degree of freedom for time and/or others often present in GAM methodology.

10. Line 194, it was also found? Here and other grammars/expressions should be checked by peer scientists in this field.

Response: We are not sure we understand the posed question. The manuscript was thoroughly checked by the authors, which are Research Scientist and Principal Investigator (Dr. Szyszkowicz), Study Coordinator (A. Lukina) and Medical Doctor (Dr. Burstein). Additionally, an independent colleague have read the paper and stated that “the paper is well written with no major revisions needed”. However, some smoothing with minor edits were suggested and the paper was corrected accordingly. We hope that this newest version will be satisfactory.

11. It is not appropriate and offers minimal knowledge by presenting the frequencies in tested associations for each exposure. Reshape this by forest plots or tables that reveals more information.

Response: We have changed the Fig 1 into three additional Figs 1, 2 and 3 that provides fully information for G40-G47. We also expanded our Supplementary Information section by adding three more Figs (S2, S3 and S4)-for G00-G99. We also expanded the results by adding RR and associated 95% CI (lines: 271-275-marked version).

6. PLOS authors have the option to publish the peer review history of their article ([what does this mean?](https://journals.plos.org/plosone/s/editorial-and-peer-review-process#loc-peer-review-history)). If published, this will include your full peer review and any attached files.


If you choose “no”, your identity will remain anonymous but your review may still be made public.


**Do you want your identity to be public for this peer review?** For information about this choice, including consent withdrawal, please see our [Privacy Policy](https://www.plos.org/privacy-policy).

Reviewer #1: No

Reviewer #2: No
