# Supplementary material for: Urban air pollution and emergency department visits related to central nervous system diseases
Source: PLoS One. 2022 Jun 27;17(6):e0270459. doi: 10.1371/journal.pone.0270459 (PMC9236246; doi:10.1371/journal.pone.0270459)
Supplement: Attachment — Submitted filename: PONE-D-21-18477R1-Rebuttal_Letter.docx [file pone.0270459.s003.docx]

Dear Dr. Manpreet Singh Bhatti,

We appreciate your time and effort in handling our manuscript entitled “Urban air pollution and emergency department visits related to central nervous system diseases” (PONE-D-21-18477R1). We also thank the reviewers for taking the time to read our manuscript and provide constructive comments. We have responded to each comment (in blue text) and have submitted both a marked and clean versions of the manuscript.

Thank you once again for considering this manuscript for publication as a Research Article in *PLOS One*. Looking forward to hearing back from you.

Kindest Regards,

Dr. Mieczysław Szyszkowicz

(new email: [mietek.szyszkowicz@hc-sc.gc.ca](mailto:mietek.szyszkowicz@hc-sc.gc.ca) and [mszyszkowicz@yahoo.ca](mailto:mszyszkowicz@yahoo.ca))

Response to Reviews:

Journal Requirements:

Comment: Please review your reference list to ensure that it is complete and correct. If you have cited papers that have been retracted, please include the rationale for doing so in the manuscript text, or remove these references and replace them with relevant current references. Any changes to the reference list should be mentioned in the rebuttal letter that accompanies your revised manuscript. If you need to cite a retracted article, indicate the article’s retracted status in the References list and also include a citation and full reference for the retraction notice.

Response: We have made some significant changes to the Reference list mostly by moving some of the references that already existed in the previous version of the paper but for more consistent flow and also by adding a few additional references as requested by one of the reviewers. References that have been moved are: #17 (old #52): Chen H, Kwong JC, Copes R. et al. Exposure to ambient air pollution and the incidence of dementia: a population-based cohort study. Environ. Int. 2017; 108: 271-277, #18 (old #53): Clifford A, Lang L, Chen R. et al. Exposure to air pollution and cognitive functioning	across the life course – a systematic literature review. Environ. Res. 2016; 147: 383-398, and # 34 (old #51): Vicedo-Cabrera AM, Sera F, Liu C. et al. Short term association between ozone and mortality: global two stage time series study in 406 locations in 20 countries. BMJ 2020, 368: m108. doi:10.1136/bmj.m108.), which made many references to shift in their consecutive numbers as well. New references that have been added are #54: Hu C, Fang Y, Li F, Dong B, Hua X, Jiang W, Zhang H, Lyu Y, Zhang X. Association between ambient air pollution and Parkinson’s disease: systematic review and meta-analysis. Environ. Res. 2019; 168: 448-459, #55: Lavery AM, Waubant E, Casper TC, Roalstad S, Candee M, Rose J, Belman A, Weinstock-Guttman B, Aaen G, Tillema J, Rodriguez M, Ness J, Harris Y, Graves J, Krupp L, Charvet L, Benson L, Gorman M, Moodley M, Rensel M, Goyal M, Mar S, Chitnis T, Schreiner T, Lotze T, Greenberg B, Kahn I, Rubin J, Waldman AT. Urban air quality and associations with pediatric multiple sclerosis. Ann. Clin. Transl. Neur. 2018; 5(10): 1146-1153, #56: Abbaszadeh S, Tabary M, Aryannejad A, Abolhasani R, Araghi F, Khaheshi I, Azimi A. Air pollution and multiple sclerosis: a comprehensive review. Neurol. Sci. 2021; 42: 4063-4072, #57: Thilakaratne RA, Malig BJ, Basu R. Examining the relationship between ambient carbon monoxide, nitrogen dioxide, and mental health-related emergency department visits in California, USA. Sci. Total Environ. 2020; 746: 140915, #58: Szyszkowicz M, Thompson EM, Colman I, Rowe BH. Ambient air pollution exposure and emergency department visits for substance abuse. PLoS ONE 2018; 13(6): e0199826, #59: Goebel U, Wollborn J. Carbon monoxide in intensive care medicine-time to start the therapeutic application. Intens. Care Med. Exp. 2020; 8:2, #60: Szyszkowicz M, Kousha T, Kingsbury M, Colman I. Air pollution and emergency department visits for depression: a multicity case-crossover study. Environ. Health Insights 2016; 10: 155-161, and #61: Lin G, Li L, Song Y, Zhou Y, Shen S, Ou C. The impact of ambient air pollution on suicide mortality: a case-crossover study in Guangzhou, China. Environ. Health 2016; 15:90.). To accompany all these new references, the Özkaynak H, Baxter LK, Burke J. Evaluation and application of alternative air pollution exposure metrics in air pollution epidemiology studies. J. Expo. Sci. Env. Epid. 2013; 23: 565 reference have been shifted to its last position as #62.

We also removed a few references, including: Kim CS, Hu SC. Regional deposition of inhaled particles in human lungs: comparison between men and women. Appl. Physiol. 1998; 84(6): 1834-1844. (#51) and Lipton RB, Mazer C, Newman LC, Solomon S. Sumatriptan relies migraine like headaches associated with carbon monoxide. Headache 1997; 37: 392-395. (#53)

Additional Editor Comments:

Nil


[Note: HTML markup is below. Please do not edit.]

Reviewers' comments:

Reviewer's Responses to Questions

**Comments to the Author**

1. If the authors have adequately addressed your comments raised in a previous round of review and you feel that this manuscript is now acceptable for publication, you may indicate that here to bypass the “Comments to the Author” section, enter your conflict of interest statement in the “Confidential to Editor” section, and submit your "Accept" recommendation.

Reviewer #1: All comments have been addressed

Reviewer #2: All comments have been addressed

Reviewer #3: (No Response)

Reviewer #4: All comments have been addressed

Reviewer #5: (No Response)

2. Is the manuscript technically sound, and do the data support the conclusions?

The manuscript must describe a technically sound piece of scientific research with data that supports the conclusions. Experiments must have been conducted rigorously, with appropriate controls, replication, and sample sizes. The conclusions must be drawn appropriately based on the data presented.

Reviewer #1: Yes

Reviewer #2: Yes

Reviewer #3: Yes

Reviewer #4: Yes

Reviewer #5: Partly

3. Has the statistical analysis been performed appropriately and rigorously?

Reviewer #1: Yes

Reviewer #2: Yes

Reviewer #3: No

Reviewer #4: Yes

Reviewer #5: No

4. Have the authors made all data underlying the findings in their manuscript fully available?

The [PLOS Data policy](http://www.plosone.org/static/policies.action#sharing) requires authors to make all data underlying the findings described in their manuscript fully available without restriction, with rare exception (please refer to the Data Availability Statement in the manuscript PDF file). The data should be provided as part of the manuscript or its supporting information, or deposited to a public repository. For example, in addition to summary statistics, the data points behind means, medians and variance measures should be available. If there are restrictions on publicly sharing data—e.g. participant privacy or use of data from a third party—those must be specified.

Reviewer #1: No

Reviewer #2: Yes

Reviewer #3: No

Reviewer #4: Yes

Reviewer #5: No

5. Is the manuscript presented in an intelligible fashion and written in standard English?

PLOS ONE does not copyedit accepted manuscripts, so the language in submitted articles must be clear, correct, and unambiguous. Any typographical or grammatical errors should be corrected at revision, so please note any specific errors here.

Reviewer #1: Yes

Reviewer #2: Yes

Reviewer #3: Yes

Reviewer #4: Yes

Reviewer #5: Yes

6. Review Comments to the Author

Please use the space provided to explain your answers to the questions above. You may also include additional comments for the author, including concerns about dual publication, research ethics, or publication ethics. (Please upload your review as an attachment if it exceeds 20,000 characters)

Reviewer #1: (No Response)

Reviewer #2: The authors have responded to my previous comments, but I have to mention that this manuscript still can be polished by editing service.

Response: We have thoroughly gone throughout the manuscript and corrected/improved sentences (all changes tracked). We hope that this new version is finally to your satisfaction.

Reviewer #3: The authors made a sincere effort in addressing the questions raised in the previous round. I have the following additional comments/clarifications:

Comment 1. A conditional quasi-Poisson regression model was used for fitting the data. Was there any count of value = 0? In such a situation, zero-augmented models need to be sued.

Response: Thank you for this question. Count value = 0 does not create the problems in the used method. We have 1008 Levels: 2004:1:1, 2004:1:2, …, 2015:12:7 – where each “year:month:day of week” may have 4 or 5 days. It may happen that for a specific stratum we have all days with 0 counts. In the used approach, it is not the problem. This situation creates problem in the standard case-crossover method, where the Cox proportional-hazards model is applied (conditional logistic regression). Before applying such models, we need to remove each stratum with all zeros, but not in here realized method.

Comment 2. The authors state: "In total, 2,160 statistical models {15 (time lags expressed as days) x 18 (strata) x 8 (air pollutants and air quality health indices)} were applied". If so many models were run, how was the best-fitting model determined (via model comparison statistics, such as AIC/BIC, etc)?

Response: We organized our data as time-series daily counts (and daily environmental factors). The model was fitted to the triple combination (i.e., stratum, air pollutant (five individual pollutants and two air quality health indices), and time lag expressed as days); therefore, we have such 2,160 statistical combinations of the triples. We do not validate (test AIC/BIC) the constructed model. In the used approach we do not have “time” as it is used in the GAM (GLM), where we need to model time variable (and validate the fitted models). Here, the constructed clusters controls for “time” – we conditioned to the sum of counts for each cluster. We added a mathematical description (lines 214-221-marked version).

Reviewer #4: The authors have responded well to the statistical issues raised in the previous review. There is no further statistical concern about this revised manuscript.

Response: Thank you for reviewing our manuscript and providing constructive comments.

Reviewer #5: Comment 1. The literature on previous studies was not properly surveyed. A thorough review is recommended.

Response: We have expanded on the discussion section and added more information on health effects associated with CO exposure and other ambient air pollutants (fine particulate matter and sulfur dioxide), the literature that was included is recent starting from 2016 (Szyszkowicz M, Kousha T, Kingsbury M, Colman I. Air pollution and emergency department visits for depression: a multicity case-crossover study. Environ. Health Insights 2016; 10: 155-161 and Lin G, Li L, Song Y, Zhou Y, Shen S, Ou C. The impact of ambient air pollution on suicide mortality: a case-crossover study in Guangzhou, China. Environ. Health 2016; 15:90.) and ending with the most recent from 2020 and 2021 (Abbaszadeh S, Tabary M, Aryannejad A, Abolhasani R, Araghi F, Khaheshi I, Azimi A. Air pollution and multiple sclerosis: a comprehensive review. Neurol. Sci. 2021; 42: 4063-4072, Thilakaratne RA, Malig BJ, Basu R. Examining the relationship between ambient carbon monoxide, nitrogen dioxide, and mental health-related emergency department visits in California, USA. Sci. Total Environ. 2020; 746: 140915., and Goebel U, Wollborn J. Carbon monoxide in intensive care medicine-time to start the therapeutic application. Intens. Care Med. Exp. 2020; 8:2.) (lines: 333-350-marked version).

Comment 2. In line number 160, the authors used the formula for generating AQHI values involving an exponential function. Would the authors justify the basis for taking exponential function? Is it possible to generate for AQHI values by using a logarithmic function?

Response: Thank you for this question. The air quality health index (or simply AQHI) is calculated as specified in the cited publication (Stieb DM et al, A new multipollutant, no-threshold air quality health index based on short-term associations observed in daily time-series analyses. J. Air Waste Manag. Assoc. 2008; 58(3):435-450. doi:10.3155/1047-3289.58.3.435). Since 2008, the AQHI is a standard scale in Canada designed for general Canadian population to understand how air quality means to their health. It is scaled as 1-10 or 10+ and is hourly reported to Canadian population. It is a preventive tool – the higher the number, the greater the health risk associated with the air quality, meaning that persons are advised to limit their outdoor activities (i.e., 1-3 mean “low health risk”, 4-6 mean “moderate health risk”, 7-10 mean “high health risk”, and 10+ mean “very high health risk”). In summary – we have not changed the definition (exp(x) vs. log(x)). It is an interesting proposition to apply “log”. We cannot modify the representation – as it will no longer be AQHI, which is an approved/accepted scale in Canada.

Comment 3. The authors should clearly explain the Generalized Non-linear Model. Is it possible to replace the ‘Quasi Poisson’ distribution by any other discrete or continuous distribution?

Response: Thank you for this question. There are a few methods, which can be used – main categories of the methods are: time-series (TS) or case-crossover (CC) designs.

In TS, daily counts are analyzed, usually generalized linear model (GLM) is used and Quasi-Poisson is applied as a link function.

In the standard CC method, individual events and matched controls period are analyzed. The conditional logistic regression is usually applied.

In the present study, we analyze daily counts (as in TS), but we are using the time-stratified CC design to control for “time” variable. The applied method and comparison with other techniques (+ software) are presented in Armstrong et al. Conditional Poisson models: a flexible alternative to conditional logistic case cross-over analysis. BMC Med. Res. Methodol. 2014; 14:122. doi: 10.1186/1471-2288-14-122.

We added some mathematical descriptions. The methodology is well represented/described in the above-cited reference.

Comment 4. The results of the statistical analysis (Table 3, Table S1, and Table S2) were not represented diagrammatically. The authors did not clearly explain the descriptive statistics. What could be concluded about the data from mean, median, and quartile values.

Response: The descriptive statistics for air pollutants used in our study are described on lines 242-253-marked version), mean, maximum and minimum levels are presented for each air pollutant (and air health index) for two seasons (warm and cold) and year round. As for supplemental materials, the ED visits (as *daily counts*) and environmental characteristics data are mainly for later publications and for other centers to compare/reference– we characterized the environmental conditions in Toronto, Canada. In addition, on the e-location already provided in the manuscript (<https://github.com/szyszkowiczm/NERVEToronto>), which contains the histograms of all used air pollutants, weather factors and the map of Toronto (population density and monitoring stations).

The results are generated in 3D space with the coordinates (stratum, air pollutant, lag – 3 axes). The figures S1, S2, and S3 are the projections on 2D – show scores of the significant results.

Comment 5. The novelty of the proposed approach has not been highlighted. Moreover, the authors did not emphasize on the significance of the proposed approach over the approaches discussed in the previous studies.

Response: We emphasised the uniqueness of the present study in introduction (lines 119-129-marked version); however, we added a bit more details of our study compared to previous studies (lines 123-125-marked version).

Comment 6. It would be better if the authors represent total frequencies (as in Figures 1, 2, 3, S1, S2, and S3) by a frequency diagram.

Response: We tabulated all the results from 2,160 models (RR and 95%CIs), also we included histogram/frequencies of the environmental factors and the map of Toronto in e-location mentioned on 227-marked version. Figure S4 and S5 are maps to the numerical results (RR, 95%CIs) for G00-G99 “all nervous system diseases” and G40-G47 “episodic and paroxysmal disorders”, respectively.

7. PLOS authors have the option to publish the peer review history of their article ([what does this mean?](https://journals.plos.org/plosone/s/editorial-and-peer-review-process#loc-peer-review-history)). If published, this will include your full peer review and any attached files.


If you choose “no”, your identity will remain anonymous but your review may still be made public.


**Do you want your identity to be public for this peer review?** For information about this choice, including consent withdrawal, please see our [Privacy Policy](https://www.plos.org/privacy-policy).

Reviewer #1: No

Reviewer #2: No

Reviewer #3: No

Reviewer #4: No

Reviewer #5: No
